# LEANING INTO COLLABORATION AT ITHACA COLLEGE

**DOI:** 10.1093/geroni/igad104.0635

**Published:** 2023-12-21

**Authors:** Mary Ann Erickson, Elizabeth Bergman

**Affiliations:** Ithaca College, Ithaca, New York, United States; Ithaca College, Ithaca, New York, United States

## Abstract

Ithaca College is a private comprehensive college of about 6,000 students in upstate New York. Its Gerontology Institute (ICGI) was formed in 1992, with a major and a minor in Aging Studies added in 2001. Despite a high profile in the local community and in the field of gerontology, program evaluation consistently revealed important challenges including trouble promoting a gerontology major to high school seniors (especially important at a tuition-dependent school like Ithaca College) and credit-heavy health professions programs where advisors were reluctant to have students add a minor or double major. Institutional forces accelerated by the pandemic led to retrenchment in 2021, including the loss of the major as well as the ICGI being folded into a department of health sciences and public health. In this presentation, we share both details of past challenges as well as the ways in which we are moving forward with a more collaborative model focused on connections within the School of Health Sciences and Human Performance.

